# Discovery of novel PDGFR inhibitors targeting non-small cell lung cancer using a multistep machine learning assisted hybrid virtual screening approach

**DOI:** 10.1039/d4ra06975g

**Published:** 2025-01-10

**Authors:** Sandhi Kranthi Reddy, S. V. G. Reddy, Syed Hussain Basha

**Affiliations:** a Department of CSE, GST, GITAM (Deemed to be University) Visakhapatnam A.P India; b Innovative Informatica Technologies Hyderabad Telangana India

## Abstract

Non-Small Cell Lung Cancer (NSCLC) is a formidable global health challenge, responsible for the majority of cancer-related deaths worldwide. The Platelet-Derived Growth Factor Receptor (PDGFR) has emerged as a promising therapeutic target in NSCLC, given its crucial involvement in cell growth, proliferation, angiogenesis, and tumor progression. Among PDGFR inhibitors, avapritinib has garnered attention due to its selective activity against mutant forms of PDGFR, particularly PDGFRA D842V and KIT exon 17 D816V, linked to resistance against conventional tyrosine kinase inhibitors. In recent years, Machine Learning has emerged as a powerful tool in pharmaceutical research, offering data-driven insights and accelerating lead identification for drug discovery. In this research article, we focus on the application of Machine Learning, alongside the RDKit toolkit, to identify potential anti-cancer drug candidates targeting PDGFR in NSCLC. Our study demonstrates how smart algorithms efficiently narrow down large screening collections to target-specific sets of just a few hundred small molecules, streamlining the hit discovery process. Employing a Machine Learning-assisted virtual screening strategy, we successfully preselected 220 compounds with potential PDGFRA inhibitory activity from a vast library of 1.048 million compounds, representing a mere 0.013% of the original library. To validate these candidates, we employed traditional genetic algorithm-based virtual screening and docking methods. Remarkably, we found that ZINC000002931631 exhibited comparable or even superior inhibitory potential against PDGFRA compared to Avapritinib, which highlights the value of our Machine Learning approach. Moreover, as part of our lead validation studies, we conducted molecular dynamic simulations, revealing critical molecular–level interactions responsible for the conformational changes in PDGFRA necessary for substrate binding. Our study exemplifies the potential of Machine Learning in the drug discovery process, providing a more efficient and cost-effective means of identifying promising drug candidates for NSCLC treatment. The success of this approach in preselecting compounds with potent PDGFRA inhibitory potential highlights its significance in advancing personalized and targeted therapies for cancer treatment.

## Abstract 2

Identified novel PDGFR inhibitor targeting non-small cell lung cancer using machine learning from vast library of 1.04 million compounds.

## Graphical abstract

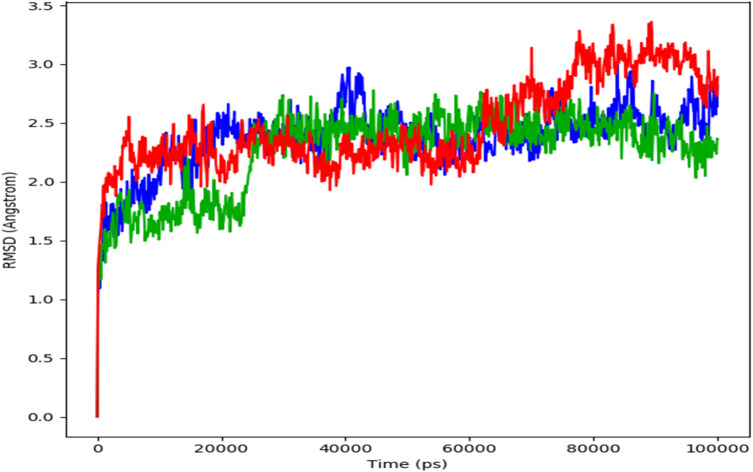

## Introduction

Non-Small Cell Lung Cancer (NSCLC) is a significant global health challenge and is the leading cause of cancer-related mortality worldwide. ^1^ Accounting for approximately 85% of all lung cancer cases, NSCLC encompasses a diverse group of malignancies arising from the epithelial cells of the lungs. It is characterized by aggressive tumor growth, early metastasis, and limited treatment options, which necessitates urgent research efforts to improve patient outcomes. ^2^ NSCLC is further classified into different subtypes, including adenocarcinoma, squamous cell carcinoma, and large cell carcinoma, each with distinct histological and molecular features. The identification and understanding of these subtypes have revolutionized the landscape of personalized medicine, as specific molecular alterations can serve as therapeutic targets and inform treatment decisions. ^3^ Smoking remains the primary risk factor for NSCLC, with the risk strongly correlated with the duration and intensity of smoking exposure. ^4^ However, in recent years, the incidence of NSCLC among non-smokers, especially in women, has been rising, suggesting that other factors, such as environmental exposure and genetic predisposition, may also contribute to its development. ^5^

Despite advances in early detection and treatment modalities, the majority of NSCLC patients are diagnosed at an advanced stage, when curative treatment options are limited. ^6^ Standard treatments include surgical resection, radiation therapy, and chemotherapy, often used in combination depending on the stage and extent of the disease. ^7^ In the last decade, targeted therapies and immunotherapies have emerged as game-changing options for subsets of NSCLC patients, offering improved survival rates and fewer side effects compared to conventional chemotherapy. ^8^ However, challenges persist, such as resistance to therapies, disease relapse, and the identification of novel targets for those without actionable mutations. Consequently, ongoing research efforts focus on identifying new therapeutic targets, understanding the mechanisms of resistance, and developing innovative treatment strategies to combat NSCLC effectively. ^9^

The Platelet-Derived Growth Factor Receptor (PDGFR) has emerged as a promising target in NSCLC due to its critical role in various cellular processes, including cell growth, proliferation, angiogenesis, and tumor progression. ^10^ PDGFR belongs to the receptor tyrosine kinase family and is activated by binding to its ligands, primarily platelet-derived growth factors (PDGFs). ^11^ Upon activation, PDGFR triggers intracellular signaling cascades, such as the MAPK and PI3K pathways, which play pivotal roles in cell survival, migration, and angiogenesis. ^12^

Increasing evidence suggests that PDGFR signaling is dysregulated in NSCLC and is associated with aggressive tumor behavior, metastasis, and therapeutic resistance. Overexpression and activation of PDGFR have been observed in NSCLC patient samples and cell lines, making it an attractive therapeutic target. ^13^ Preclinical studies investigating the inhibition of PDGFR signaling in NSCLC have demonstrated promising results. ^14^ Inhibitors targeting PDGFR have shown antitumor activity and suppressed tumor growth in experimental models of NSCLC. ^15^ Moreover, PDGFR inhibitors have demonstrated the potential to enhance the efficacy of existing therapies and overcome resistance to conventional treatments, making them an appealing addition to current treatment regimens. ^16^

Clinical trials evaluating PDGFR-targeted therapies in NSCLC patients have shown encouraging outcomes, further supporting the potential utility of PDGFR inhibition in the clinical setting. ^17^ Among the PDGFR inhibitors, avapritinib has garnered increasing attention for its potent and selective activity against mutant forms of PDGFR, particularly PDGFRA D842V and KIT exon 17 D816V, which are associated with resistance to conventional tyrosine kinase inhibitors. ^18^

Avapritinib is an orally available small molecule kinase inhibitor developed by Blueprint Medicines Corporation. It is designed to specifically target and inhibit the activity of PDGFR and KIT receptors, which play critical roles in cell signaling and cancer development. Avapritinib is specifically intended for adults with unresectable or metastatic gastrointestinal stromal tumor (GIST). ^19^ Avapritinib's exceptional specificity for PDGFR and KIT mutant forms makes it a promising candidate for targeted therapy in NSCLC patients harboring these mutations. ^20^ Preclinical studies have demonstrated avapritinib's efficacy in inhibiting tumor growth and reducing angiogenesis in NSCLC models expressing PDGFRA D842V and KIT exon 17 D816V mutations. ^21^ Moreover, avapritinib's ability to overcome resistance to other tyrosine kinase inhibitors provides a unique advantage in treating NSCLC patients with limited therapeutic options, as a repurposed drug. ^22^ Clinical trials evaluating avapritinib in NSCLC patients with PDGFR alterations have shown promising results, with significant antitumor activity and improved overall survival rates. Notably, avapritinib's targeted approach minimizes off-target effects on normal cells, potentially reducing treatment-related toxicities compared to traditional chemotherapies. ^23^ Despite these encouraging findings, challenges remain in understanding the optimal patient selection, defining the appropriate combination therapies, and addressing potential resistance mechanisms.

In recent years, the convergence of computational approaches and molecular biology has led to groundbreaking advancements in drug discovery, particularly in the identification of potential anti-cancer agents. Machine Learning, a subset of artificial intelligence, has emerged as a powerful tool in the pharmaceutical industry for lead identification, offering accelerated and data-driven insights into novel drug candidates. Coupled with RDKit, a widely-used open-source cheminformatics toolkit, ^24^ these methodologies hold immense promise in the quest for more effective and targeted therapies for complex diseases like non-small cell lung cancer (NSCLC) through PDGFR targeting.

Machine Learning offers the advantage of processing large-scale datasets, uncovering intricate patterns, and predicting molecular properties with remarkable accuracy. In the context of lead identification, Machine Learning models are trained using diverse chemical libraries and biological data to discern molecular features associated with desired therapeutic activities. ^25^ These models can then be applied to screen vast chemical databases to prioritize promising drug candidates, accelerating the drug discovery process and reducing the associated costs and time. RDKit, on the other hand, provides a versatile and efficient toolkit for cheminformatics, allowing researchers to manipulate chemical structures, calculate molecular descriptors, and perform virtual screening and structure–activity relationship (SAR) analysis. Its seamless integration with Machine Learning workflows facilitates the rapid exploration of chemical space and the generation of predictive models that lead to rational drug design.

In this research paper, we focus on the application of Machine Learning and RDKit in lead identification for anti-cancer drug discovery, with a specific emphasis on PDGFR targeting in NSCLC, as a preliminary step towards repurposing existing PDGFR inhibitor designed for GIST as a NSCLC inhibitor along with finding similar or better molecules.

## Methods

### Molecular fingerprints

Molecular fingerprints are numerical representations of molecules that encode molecular fragments or features as binary digits (0 or 1), indicating the presence or absence of a specific feature.

#### Molecular fingerprints and their purpose

Molecular fingerprints are a type of molecular descriptors that encode molecular features as binary digits. Features present in a molecule are represented by 1, while absent features are represented by 0. Molecular fingerprints provide a numerical representation of molecules, which enables various computational analyses and modeling techniques. They are used for structure similarity searches, virtual screening of compounds, and developing predictive models like quantitative structure–activity relationship (QSAR) or quantitative structure–property relationship (QSPR) models. ^26^

Molecular fingerprints were generated using the open-source cheminformatics library, RDKit. The required libraries for the research are imported, including pandas, numpy, matplotlib, and RDKit. Pandas is used for reading CSV files, numpy for converting RDKit objects to number arrays, matplotlib for plotting, and RDKit for molecular fingerprint generation.

#### Data collection

A dataset containing molecular structures (SMILES strings) values is imported using pandas. The SMILES column from the dataset is used to generate molecular graphs using the RDKit library. The function “add_molecule_column_to_frame” from the “PandasTools” module is employed to add a new column with the molecular graphs.

#### Visualization of molecular structures

The molecular structures are displayed using the RDKit library's “Draw.MolsToImage” function. Molecular Fingerprint Generation: The research focuses on generating two molecular fingerprints available in RDKit: MACCS and 2D pharma fingerprints. For each fingerprint type, the specific RDKit functions are used to calculate the fingerprints for a given molecule.

### Protein–ligand structures visualization

The visualization software used for this study included Schrodinger's Maestro visualization program v9.6 ^27^ and Biovia Discovery Studio v16.1. ^28^ These programs were employed to visualize the receptors and ligand structures, analyze the hydrogen bonding network, calculate bond lengths, and generate images.

### Protein and ligands retrieval

The Avapritinib compound (DrugBank accession number DB15233) was obtained from the DrugBank database, ^29^ drug like molecules database of about 10 lakhs and 48 thousand compounds were retrieved from ZINC database ^30^ and the crystal structure of Platelet-derived growth factor receptor α (PDGFRA) [PDB: 5K5X] ^31^ was retrieved from the Protein Data Bank (PDB). ^32^

### Clustering

Clustering is a data analysis method that groups similar data points together, useful for pattern recognition and simplifying complex datasets. It's unsupervised and applied in various fields, but selecting the right algorithm and number of clusters can be challenging. Evaluation metrics help assess cluster quality, and visualization techniques aid in understanding the results.

The *K*-Means clustering algorithm was chosen for its efficiency and ability to handle high-dimensional, unlabelled data. *K*-Means assigns data points or compounds to clusters where points or compounds within each cluster are more similar to each other than to those in other clusters. *K*-Means offers faster computation through its iterative process when compared to other clustering algorithms Hierarchical Clustering and DBSCAN, making it ideal for high-throughput virtual screening.

We can integrate the *K*-Means clustering machine learning algorithm into the elbow approach. A well-liked unsupervised machine learning approach called *K*-Means clustering is used to divide a dataset into a number of separate, non-overlapping clusters. To organize data points into *K* clusters, where *K* is a predetermined number, is the basic goal of *K*-Means. The goal is to reduce the sum of squared distances between data points and their cluster's mean. Each data point belongs to the cluster with the closest mean (center). The elbow method is utilized in *K*-Means clustering. *K*-Means doesn't need labeled data, in contrast to supervised learning. *K* cluster centroids are iteratively adjusted until they cease moving after being initialized randomly. For a better understanding, let's go over the steps involved in *K*-means clustering:

• Choose (*K*) how many clusters there should be in the dataset.

• Decide on *K* centroids at random from the dataset.

• To create *K* clusters, we will now utilize the Euclidean distance or Manhattan distance as the metric to determine the distance between each point and the nearest cluster centroid.

• Now identify the new centroid of the resulting clusters.

• Repeat step 4 after once more reassigning the entire data point based on this new centroid. The process will be repeated until the centroid's position stays the same, or there is no longer any convergence, after a predetermined number of iterations.

The key to this approach is determining the ideal number of clusters. The Elbow Method is a frequently employed technique for determining the ideal *K* value. In the Elbow method, we are actually varying the number of clusters (*K*) from 1–20 depending upon the dataset. For each value of *K*, we are calculating WCSS (Within-Cluster Sum of Square). WCSS is the sum of the squared distance between each point and the centroid in a cluster. When we plot the WCSS with the *K* value, the plot looks like an Elbow. As the number of clusters increases, the WCSS value will start to decrease. WCSS value is largest when *K* = 1. When we analyze the graph, we can see that the graph will rapidly change at a point and thus creating an elbow shape. From this point, the graph moves almost parallel to the *X*-axis. The *K* value corresponding to this point is the optimal value of *K* or an optimal number of clusters. When referring to the quality of clusters in unsupervised clustering methods like *K*-Means, the term “silhouette” in data analysis and clustering typically refers to the silhouette coefficient or silhouette score. When compared to the closest neighboring cluster, the silhouette score indicates how similar each data point in a cluster is to its neighbors in that cluster. From −1 to 1, better results are indicated by higher values.

After generating molecular fingerprints, the application of the *K*-Means clustering algorithm is followed by validation to determine the primary cluster, with a focus on identifying lead compounds exhibiting the highest binding energy.

### Deep learning

Deep learning is a subset of machine learning that uses neural networks with many layers to learn and make predictions from data. It's known for its ability to automatically extract features from raw data, making it suitable for tasks like image and speech recognition, natural language processing, and autonomous driving. Deep Convolutional Neural Networks (DCNNs) are a type of artificial neural network recognized for their autonomous capability to extract features from data. They are particularly valuable in tasks involving images, such as image recognition and computer vision. In the domain of drug discovery, DCNNs are indispensable for the analysis of molecular structures, virtual screening, and the prediction of properties. They facilitate the identification of potential drug candidates and the exploration of their interactions with biological targets, revolutionizing the drug discovery process through data-driven approaches.

A Convolutional Neural Network (CNN) is structured with several layers, including the input layer, Convolutional layer, Pooling layer, and fully connected layers. ^34^

#### Input layer

This layer receives the raw data, such as an image or sequence, and passes it forward for processing.

#### Convolutional layers

These layers apply filters (also known as kernels) to the input data to detect patterns and features. Convolutional layers are crucial for identifying shapes, edges, and more complex structures in images or sequences. ^34^

#### Pooling layers

After convolutional layers, pooling layers down sample the feature maps created by convolution, reducing the spatial dimensions. This helps reduce computational complexity and retain essential features.

#### Fully connected layers

These layers connect each neuron to all neurons in the previous layer. Fully connected layers are often used in the final stages of the network for tasks like classification or regression.
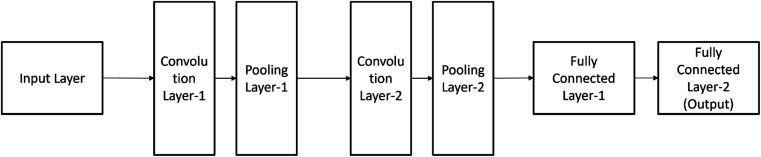


These layers work together in a hierarchical manner, with deeper layers learning increasingly abstract and complex features. DCNNs are particularly well-suited for tasks like image recognition and computer vision due to their ability to automatically learn and represent hierarchical features within data. *K*_DEEP_ is a protein–ligand affinity predictor based on DCNNs (Deep Convolutional Neural Networks) is used for the virtual screening process. ^33^ The results for the core test set for the standard PDBbind (v. 2016) ^34^ are state-of-the-art, with a Pearson's correlation coefficient of 0.82 and an RMSE in pK units between experimental and projected affinity of 1.27. *K*_DEEP_ is made available through https://PlayMolecule.org so users can quickly test their own protein–ligand complexes. Each prediction only takes a brief amount of time. ^35^*K*_DEEP_ is already in use as a desirable scoring function for contemporary computational chemistry pipelines because of its speed, performance, and simplicity. Deep Convolution Neural Networks utilized for the protein–ligand binding affinity predictions as part of the *K*_DEEP_ program voxelized given compound structure into 8 different pharmacophoric-like features (hydrophobic, aromatic, hydrogen-bond donor and acceptor, positive and negative ionizable, metallic and total excluded volume). Then, it is used as input for a DCNN model, which is pre-trained using the PDBbind v.2016 database, to predict the binding poses and binding energies.

### Molecular docking

For the semi-flexible docking studies between PDGFRA, Avapritinib and ZINC000002931631 compound, Autodock 4.0 ^36^ was the preliminary docking program employed. The ligands and protein receptors were prepared in pdbqt file format, and the size of the grid box was determined using Auto-Dock Tools version 1.5.6. The grid box, measuring 126 Å (*x*, *y*, and *z*), was set to score energy and was centered at *X* 0.024 *Y* 0.771 and *Z* −1.118 with 0.375 angstroms grid points spacing. ^37,38^

### Molecular dynamic simulations

For molecular dynamic simulation studies, Schrodinger's Desmond module v3.6 ^39^ was employed using the default protocol as explained elsewhere in detail. ^40–45^ The OPLS 2005 force field ^46^ was used along with simulation TIP3P water models. ^47^ The simulation boxes were created with periodic boundary conditions, buffered at 10 Å distances, and the volumes were calculated to be 400 000 cubic Å s for PDGFRA in its apo state, complexed with Avapritinib and in complex with ZINC000002931631 at its binding site. During the equilibration process, van der Waals and short-range electrostatic interactions were cut off at 9 Å, while long-range electrostatic interactions were computed using the Particle Mesh Ewald method. ^48^ A RESPA integrator ^49^ with a time step of 2 fs was used, and long-range electrostatics were computed every 6 fs. The equilibration was carried out using the Desmond program in the NPT ensemble ^50^ at a temperature of 300 K and 1 bar pressure using the Nose–Hoover chain relaxation thermostat method along with the Martyna–Tobias–Klein relaxation Barostat method ^51^ with isotropic coupling style at 1 ps & 2 ps timescales, respectively. Throughout the simulated timescale, all the simulations were conducted under the same temperature, pressure, and volume conditions. ^52,53^ As part of the simulation quality analysis, it was observed that the average total energy of the simulated system remained constant at −100500 kcal mol^−1^ in all cases of simulations.

### Similarity search

#### Similarity metrics ^54^

Similarity metrics, also known as similarity measures or distance metrics, are mathematical tools used to quantify the similarity or dissimilarity between two objects, datasets, or entities. These metrics play a fundamental role in various fields, including data analysis, machine learning, information retrieval, recommendation systems, and more. The choice of similarity metric depends on the nature of the data and the specific problem you're trying to solve. Here are some commonly used similarity metrics.

#### Euclidean distance ^55^

Euclidean distance measures the straight-line distance between two points in Euclidean space. It is often used for continuous data and is suitable for cases where the data features have a clear geometric interpretation. The formula for Euclidean distance between two points A and B in n-dimensional space is:
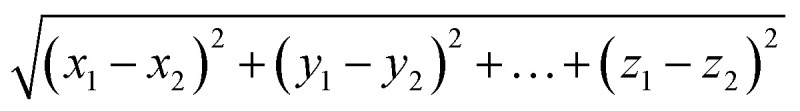


#### Cosine similarity ^56^

Cosine similarity measures the cosine of the angle between two vectors in multidimensional space. It is often used for text data or other high-dimensional data where magnitude matters less than the direction. Cosine similarity ranges from −1 (perfect dissimilarity) to 1 (perfect similarity), with 0 indicating orthogonality. The formula for cosine similarity between vectors A and B is:
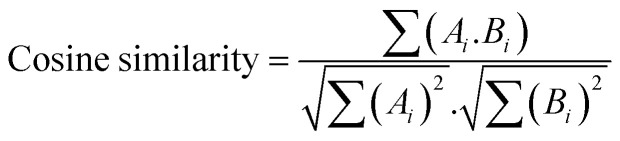


#### Jaccard similarity or tanimoto coefficient ^57^

Jaccard similarity measures the similarity between two sets by comparing the intersection of the sets to their union. It is commonly used for binary data or data with binary attributes, such as text documents represented as sets of words. The formula for Jaccard similarity is:
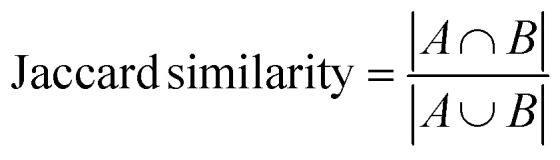


The Tanimoto coefficient, also known as the Jaccard similarity coefficient, is considered efficient for certain types of similarity comparisons, particularly in cases where binary data or binary feature vectors are involved. Here's why the Tanimoto coefficient is considered efficient: Simplicity: The Tanimoto coefficient is straightforward to compute. It measures the similarity between two sets by comparing the size of their intersection to the size of their union. This simplicity makes it computationally efficient, especially when dealing with large datasets.

#### Binary data

**Fig. 1:**
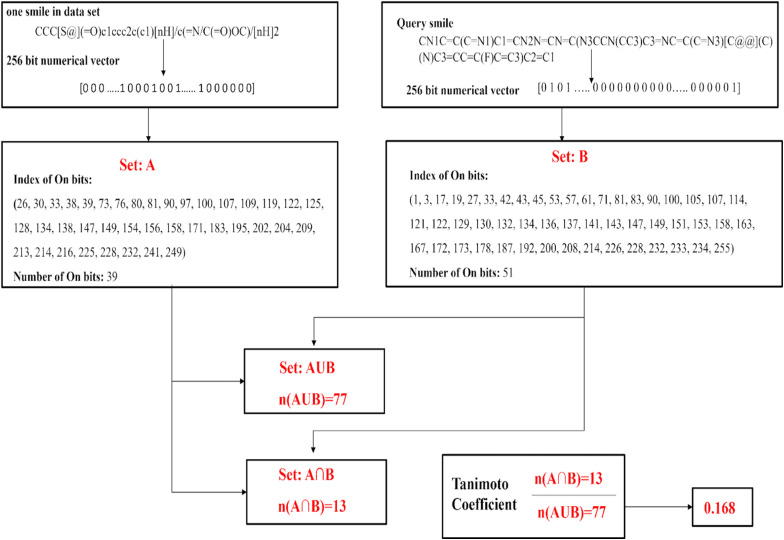
Schematic representation of Tanimoto coefficient calculation for SMILES in a dataset with respect to a query SMILES.

The Tanimoto coefficient is particularly well-suited for binary data, where each data point is represented as a binary feature vector. This is common in various applications, such as text document analysis (where words are binary features), molecular fingerprints in cheminformatics (presence/absence of substructures), and collaborative filtering in recommendation systems (user–item interactions). Sparsity: In scenarios with binary data, feature vectors are often sparse, meaning that most feature values are zero. The Tanimoto coefficient efficiently handles sparse data because it only considers the presence (1) or absence (0) of features. Set-Based Comparison: The Tanimoto coefficient is inherently a set-based similarity measure. It treats data points as sets of features, making it suitable for applications where set-like relationships are important, such as finding similar items or documents. Studies say that tanimoto coefficient is the most efficient similarity metric to find the similarity. For every smile in the dataset and for query smiles Morgan fingerprint data is generated as a binary numerical vector. From numerical vector number of On bits and On bit indexes are identified *i.e.*, the bit index where binary vector contains 1 for both smile in dataset and for query smile, union and intersection of two bits are calculated to calculate tanimoto coefficient as show in Fig. 1.

#### Structural fingerprints ^58^

These fingerprints encode the structural information of a molecule. They are typically binary or integer vectors that represent the presence or absence of specific structural features or substructures within a molecule. Common structural fingerprints include: ECFP (Extended Connectivity Fingerprints): these fingerprints encode molecular substructures by considering atom connectivity within a specified radius from each atom in the molecule. MACCS Keys: the MACCS (Molecular ACCess System) keys ^59^ are a set of 166 predefined structural keys that represent various chemical features, such as functional groups and substructures. Descriptor-based Fingerprints: these fingerprints encode the molecular properties of a compound as numerical descriptors. These descriptors can be based on various molecular properties, such as quantum chemical calculations, physical–chemical properties, or pharmacophore features. Common descriptor-based fingerprints include: physicochemical descriptors: these include properties like molecular weight, polar surface area, log *P* (partition coefficient), and many others that describe the physical and chemical characteristics of a molecule. Quantum chemical descriptors: these are derived from quantum mechanical calculations and provide detailed electronic and structural information about a molecule. Pharmacophore Fingerprints: these describe the molecular features necessary for a compound to interact with a biological target, such as protein–ligand interaction patterns. Molecular fingerprints are used in a wide range of applications, including drug discovery (virtual screening and QSAR modeling), similarity searching, clustering, and cheminformatics. In hybrid screening we have used Morgan fingerprint and 2D pharmacophore fingerprint are used because they are useful for similarity searching and molecular clustering due to their ability to capture local structural information.

**Fig. 2:**
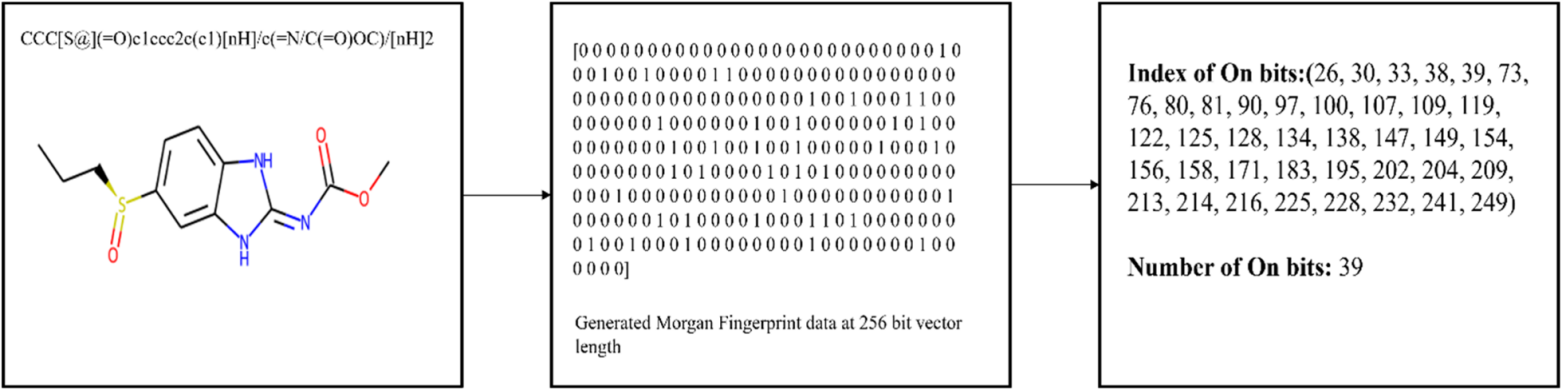
Schematic representation of Morgan Fingerprint data generation.

Morgan fingerprints, ^60^ often referred to as Morgan circular fingerprints or simply Morgan fingerprints, are a type of molecular fingerprint used in cheminformatics and computational chemistry. These fingerprints are a variation of the Extended Connectivity Fingerprints (ECFP) and are designed to represent the structural features and connectivity patterns of molecules in a binary format as shown in Fig. 2.

**Fig. 3:**
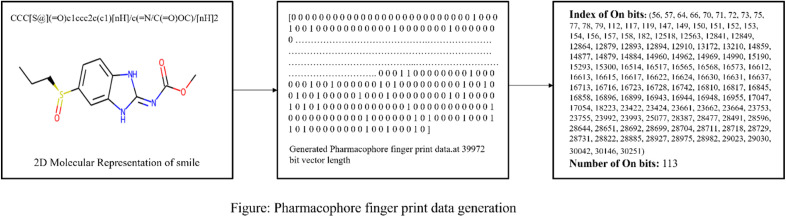
Schematic representation of Pharmacophore fingerprint data generation.

Pharmacophore fingerprints ^61–63^ are a type of molecular fingerprint used in cheminformatics and drug discovery. They are designed to capture the essential features of a molecule that are critical for its interaction with a biological target, such as a protein or enzyme as shown in Fig. 3. Pharmacophore fingerprints are widely used for ligand-based virtual screening, similarity searching, and pharmacophore modeling.

Here are some key points about pharmacophore fingerprints.

#### Pharmacophore features

Pharmacophore fingerprints encode specific chemical features or groups within a molecule that play a crucial role in its biological activity. These features can include hydrogen bond donors, hydrogen bond acceptors, aromatic rings, positive/negative ionizable groups, and hydrophobic regions.

#### Feature encoding

Each pharmacophoric feature is encoded in the fingerprint, often as binary values (1 for presence, 0 for absence) or with more detailed encoding schemes that include information about the feature type and spatial arrangement.

#### Spatial information

Pharmacophore fingerprints can capture not only the presence of features but also their spatial relationships. For example, they can encode the relative distances or angles between features within the molecule.

## Hybrid screening method- integrating clustering and deep learning for virtual screening

**Fig. 4:**
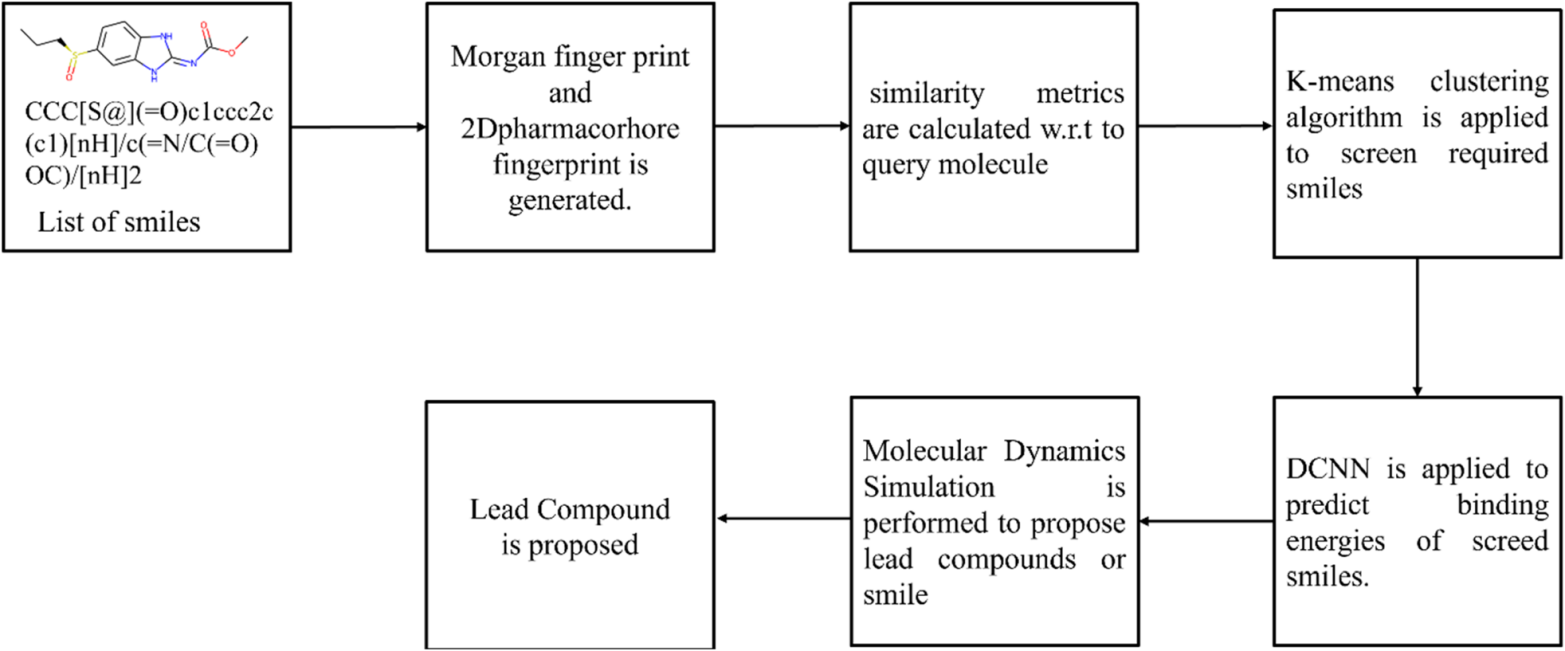
Schematic representation of hybrid screening employing *K*-Means clustering and DCNN.

**Fig. 5:**
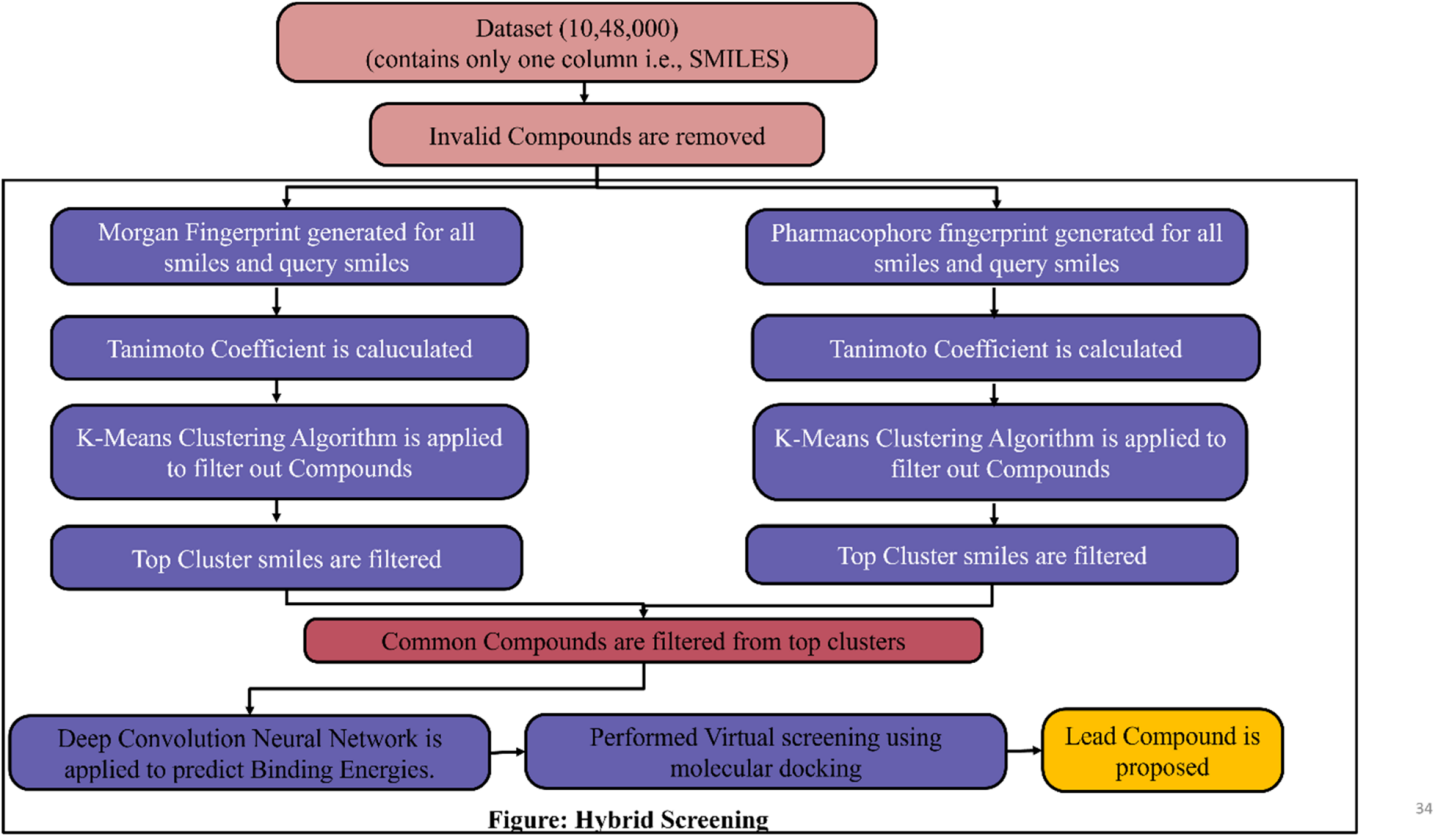
Schematic representation of hybrid screening protocol followed for the current study.

FDA has approved avapritinib for gastrointestinal stromal tumors (GIST), but for lung cancer there is no PDGFR inhibitor. Now the target is to find the new drug for non-small cell lung cancer which shows better results than PDGFR inhibitor Avapritinib by applying machine learning/deep learning techniques in lead identification and lead optimization. In lead identification, a lead compound needs to be identified for target (PDGFR). For that a dataset of drug-like molecules was downloaded from https://zinc12.docking.org/subsets/clean-drug-like which contains 10 lakhs, 48 thousand compounds/molecules. To identify the top lead compound a Hybrid screening is proposed as shown in Fig. 4 and 5. The process of Hybrid screening is as follows:

• Molecular fingerprint is calculated for every molecule or smile in dataset two molecular fingerprint data *i.e.*, Morgan finger print and 2Dpharmacorhore fingerprint is generated.

• Similarity metrics are used to find similarity between query molecule and list of compounds.

• *K*-Means Clustering algorithm is applied to filtered out required compounds.

• Convolution Neural Network(*K*-Deep) is applied on filtered lead compounds to predict Binding Energy.

• Finally Molecular dynamics simulation is performed to propose lead compounds.

The main advantage of Hybrid screening is that the time required for screening the compounds is drastically reduced because traditional virtual screening takes 100 times more than hybrid method to screen compounds and it will become standard method to identify the novel inhibitor for any disease.

Molecular fingerprint data refers to a representation of a molecule's structure and properties in a format that can be used for various computational and analytical purposes. These fingerprints are essential in the fields of chemistry, bioinformatics, and drug discovery, among others. There are two primary types of molecular fingerprints: the Morgan fingerprint and the Pharmacophore fingerprint.

## Results & discussion

**Fig. 6:**
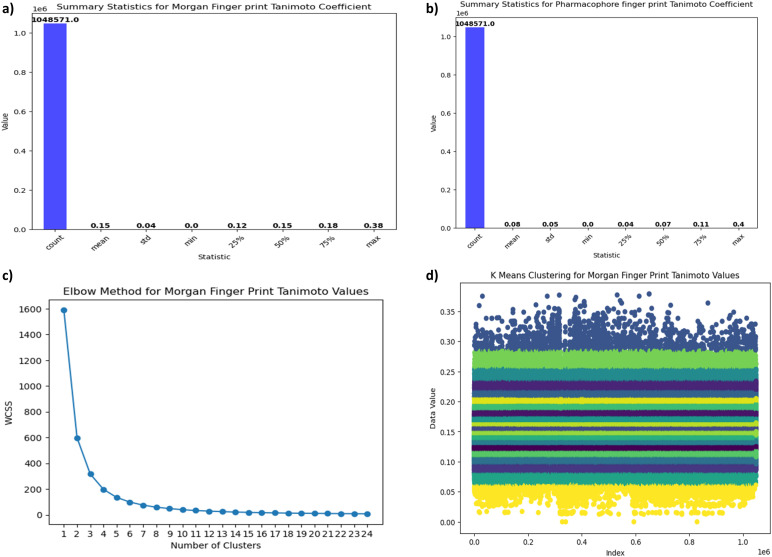
(a) Statistical overview of Tanimoto coefficients using Morgan Fingerprints. This figure displays key statistical metrics, including mean, median, standard deviation, min–max range, and percentiles, offering insights into dataset similarity based on Morgan Fingerprints. (b) Statistical overview of Tanimoto coefficients using Pharmacophore Fingerprints. This figure displays key statistical metrics, including mean, median, standard deviation, min–max range, and percentiles, offering insights into dataset similarity based on Pharmacophore Fingerprints. (c) Elbow Method for Morgan Fingerprints Clustering. This graph demonstrates the Elbow Method, a tool for determining the optimal number of clusters in Morgan Fingerprints analysis. (d) *K*-Means Clustering of Morgan Fingerprints Based on Tanimoto Values. A visual representation of dataset clustering achieved through *K*-Means clustering using Morgan Fingerprints and their Tanimoto values.

The following Fig. 6a and b provides a comprehensive overview of Tanimoto coefficient statistics, which quantify the similarity between compounds, for both the Morgan fingerprint and the Pharmacophore fingerprint. This analysis offers valuable insights into the degree of structural similarity or dissimilarity among compounds represented by these two distinct fingerprinting methods, contributing to a deeper understanding of their relationships.

**Fig. 7:**
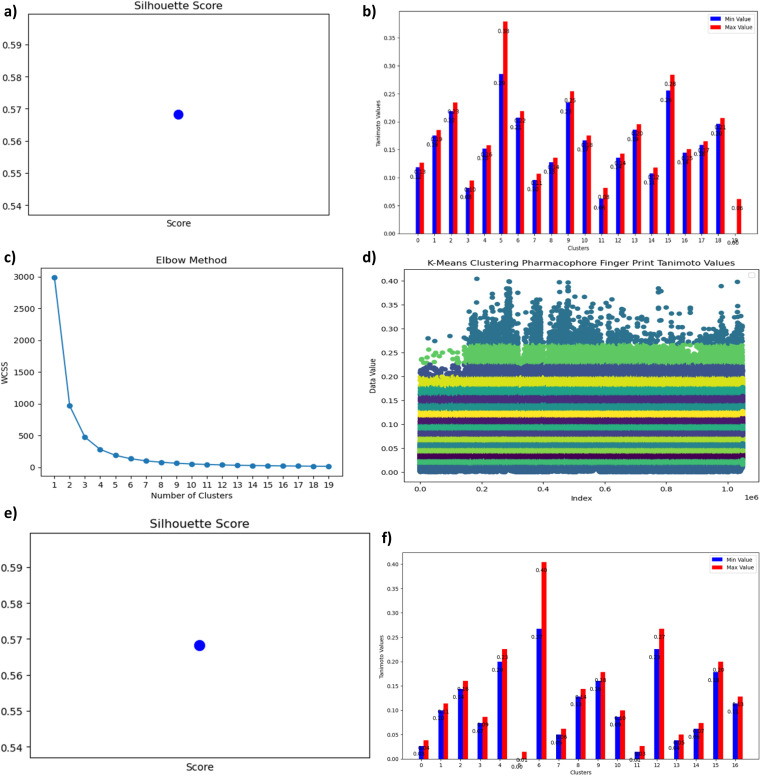
(a) A graphical representation illustrating the Silhouette Score, a vital metric for validating *K*-Means clustering. (b) Bar Chart Depicting the Range of Minimum and Maximum Values Across All 20 Clusters. (c) Elbow Method for Pharmacophore fingerprint Clustering. This graph demonstrates the Elbow Method, a tool for determining the optimal number of clusters in Pharmacophore Fingerprint analysis. (d) *K*-Means Clustering of Pharmacophore fingerprints Based on Tanimoto Values. A visual representation of dataset clustering achieved through *K*-Means clustering using Pharmacophore fingerprint and their Tanimoto values. (e) A graphical representation illustrating the Silhouette Score, a vital metric for validating *K*-Means clustering. (f) Bar Chart Depicting the Range of Minimum and Maximum Values across all 17 Clusters.

Now *K*-Means Clustering algorithm is applied for Tanimoto values of morgan and pharmacophore to filter out top most smiles. The elbow method is applied on morgan finger Tanimoto values, from which can we conclude the number of clusters. The following Fig. 6c represents the elbow method: *x* axis represents number of clusters and *y* axis represents WCSS. From the graph we can observe that there is a constant flow from cluster 18 to 22, hence we can consider the number of clusters as 20. Now *K*-Means algorithms are applied on morgan Tanimoto values for *K* value as 20. After applying *K*-Means clustering on Morgan Fingerprint Tanimoto values the following clusters (shown in Fig. 6d) are generated. The following Fig. 7a shows the silhouette score of above clustering as 0.57 which is considered as efficient clustering. The cluster number, count, minimum and maximum values are shown in Table 1 as well as in Fig. 7b. By observing the following table and graph the sixth cluster *i.e.*, cluster no:5 has the highest range of Tanimoto values containing values between 0.29 to 0.38 with entries 2197 which are useful for further screening process.

**Table 1:** Cluster statistics – comprehensive data including cluster number, count of elements, minimum value, and maximum value, across a total of 20 distinct clusters

Cluster	Count	Min	Max
0	84 622	0.12	0.13
1	73 855	0.18	0.19
2	26 780	0.22	0.23
3	39 151	0.08	0.1
4	73 587	0.15	0.16
5	2197	0.29	0.38
6	34 411	0.21	0.22
7	59 637	0.1	0.11
8	83 599	0.13	0.14
9	16 624	0.23	0.25
10	87 920	0.17	0.18
11	21 203	0.06	0.08
12	91 496	0.14	0.14
13	59 584	0.19	0.2
14	76 334	0.11	0.12
15	7938	0.26	0.28
16	85 612	0.14	0.15
17	76 067	0.16	0.17
18	42 065	0.2	0.21
19	5889	0	0.06

Similarly *K*-Means applied for pharmacophore fingerprint data before that number of clusters need to be identified based on the Elbow method. The following Fig. 7c represents the elbow method: *x* axis represents number of clusters and *y* axis represents WCSS. From the graph it is observed that there is a constant flow from the number of clusters from 15 to 19, hence we can consider the number of clusters as 17. Now *K*-Means algorithms are applied on pharmacophore Tanimoto values for *K* value as 17. The following graph shows the clustering of pharmacophore Tanimoto values (Fig. 7d).

When referring to the quality of clusters in unsupervised clustering methods like *K*-Means, the term “silhouette” in data analysis and clustering typically refers to the silhouette coefficient or silhouette score. When compared to the closest neighboring cluster, the silhouette score indicates how similar each data point in a cluster is to its neighbors in that cluster. From −1 to 1, better results are indicated by higher values. The following Fig. 7e shows the silhouette score of above clustering as 0.57 which is considered as efficient clustering.

*K*-Means clustering algorithm generated twenty clusters for given data, the cluster number, count, minimum and maximum values are shown in the Table 2 as well as in graph Fig. 7f. By observing the following table and graph the sixth cluster *i.e.*, cluster no: 6 has the highest range of Tanimoto values containing values between 0.27 to 0.40 with entries 1958 which are useful for further screening process.

**Table 2:** Cluster statistics – comprehensive data including cluster number, count of elements, minimum value, and maximum value, across a total of 17 distinct clusters

Cluster	Count	Min	Max
0	93 397	0.03	0.04
1	73 569	0.1	0.11
2	43 931	0.14	0.16
3	89 486	0.07	0.09
4	18 249	0.2	0.23
5	86 610	0	0.01
6	1958	0.27	0.4
7	93 278	0.05	0.06
8	53 014	0.13	0.14
9	35 441	0.16	0.18
10	82 135	0.09	0.1
11	92 748	0.01	0.03
12	9554	0.23	0.27
13	93 130	0.04	0.05
14	92 014	0.06	0.07
15	26 909	0.18	0.2
16	63 148	0.11	0.13

With the help of *k*-means clustering algorithms two clusters are identified, one from Morgan Fingerprint *i.e.* cluster number 06 that contains 2197 smiles and other one is from pharmacophore fingerprint *i.e.*, cluster number 07 that contains 1958 smiles as shown in Table 3.

**Table 3:** Fingerprint overview – fingerprint names, ranges, cluster assignments, and no of Entry

S.No	Name of the finger print	Range	Number of smiles	Cluster number
1	Morgan fingerprint	0.29 to 0.38	2197	06
2	Pharmacophore fingerprint	0.27 to 0.40	1958	07

**Fig. 8:**
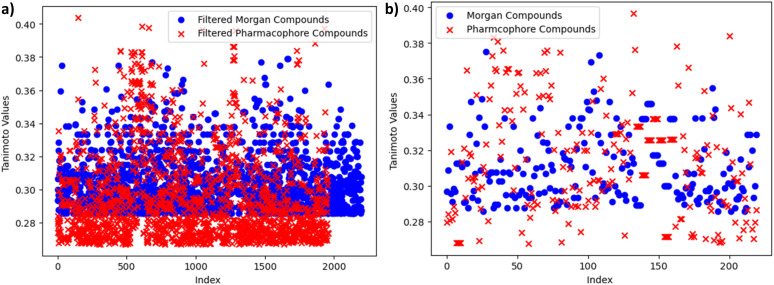
(a) Comparative Tanimoto similarity analysis of Morgan and Pharmacophore clusters. (b) Comparative common smiles Tanimoto similarity analysis of Morgan and Pharmacophore.

The graph, presented in Fig. 8a, provides a comparative analysis of Tanimoto similarity values between two distinct clusters: Morgan and Pharmacophore. The horizontal axis represents the index, sequentially identifying data points, while the vertical axis quantifies the Tanimoto similarity values. This visualization offers insights into the degree of similarity or dissimilarity between compounds within these clusters, aiding in the assessment of their structural relationships.

In this comparative analysis, we have examined two distinct clusters of chemical compounds. The first cluster comprises 2197 entries, each characterized by their SMILES representations and associated Tanimoto similarity values. The second cluster consists of 1958 entries, exclusively associated with the Pharmacophore fingerprint category. Importantly, a subset of common SMILES entries was identified from both the Morgan and Pharmacophore fingerprint datasets which is shown in Table 4 and Fig. 8b. The motivation behind this selection was to pinpoint the most relevant compounds, particularly those with potential for high binding affinity or biological activity. By focusing on these shared SMILES entries, this analysis seeks to highlight and prioritize compounds of significant interest for further exploration and investigation, potentially accelerating the process of identifying promising candidates in chemical and drug discovery research.

**Table 4:** Fingerprint overview – fingerprint names, ranges, number of common smiles from two clusters

S. no	Finger print	Range (min & max)	Number of common smiles
1	Morgan fingerprint	0.29 to 0.38	220
2	Pharmacophore fingerprint	0.27 to 0.40	

Traditional virtual screening methods often require a significant amount of time to sift through large datasets, typically reducing compounds from hundreds of thousands to just thousands. However, the integration of similarity metrics and machine learning has revolutionized this process, significantly reducing the time required to filter compounds to a minimum.

### Virtual screening

Following the fingerprint-based screening described above, a total of 220 commonly identified compounds were subjected to virtual screening using a computational pipeline. The results of this virtual screening revealed that all 220 compounds exhibited successful docking with the PDGFR protein, displaying a binding energy range spanning from −10.7 to −7.4 Kcal mol^−1^. In comparison, the control compound, avapritinib, exhibited a binding energy of −9.7 Kcal mol^−1^.

The comprehensive list of compounds resulting from the virtual screening process can be found in the supplementary material. Among the 220 compounds evaluated, we have identified the top 30 compounds, which, along with the control compound avapritinib, displayed a binding affinity of −9.8 Kcal mol^−1^ or higher during molecular docking studies. These top-ranking compounds have been selected for further in-depth evaluation.

### Molecular docking

Among the Top 30 compounds evaluated, we have found that all 30 compounds exhibited successful docking with the PDGFR protein, displaying a binding energy range spanning from −10.69 to −7.65 Kcal mol^−1^ and rest of the compounds showed −8.98 Kcal mol^−1^ of less in binding affinity towards PDGFR.

According to the docking results, Avapritnib was found to be docked at the binding site with a binding energy of −10.69 kcal mol^−1^ and a predicted IC50 value of 14.49 nM (nanomolar). Whereas, ZINC000002931631 successfully bound to the PDGFRA binding site, occupying the available space with a binding energy of −10.58 kcal mol^−1^ and a predicted IC50 value of 17.60 nM (nanomolar), suggesting that our proposed compound ZINC000002931631 is very close to the inhibitory potential of Avapritinib.

**Fig. 9:**
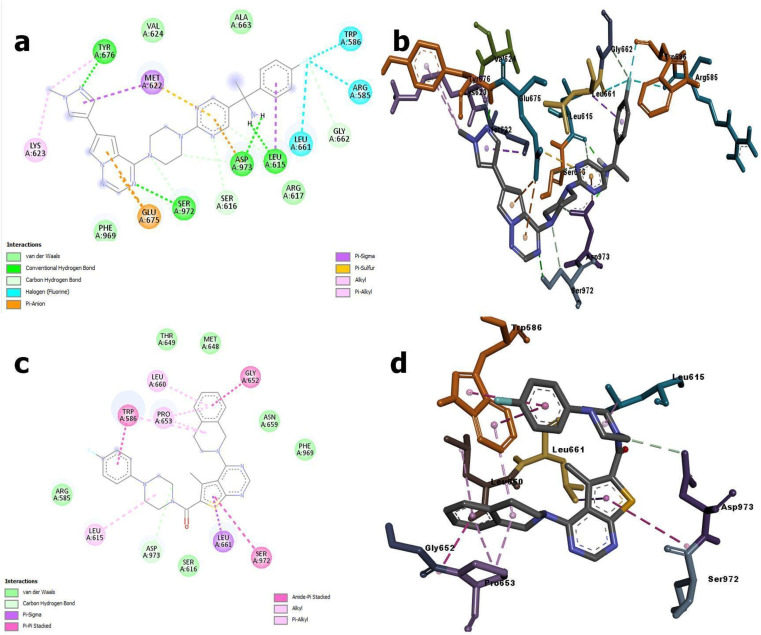
Molecular interactions of Avapritinib and ZINC000002931631 compounds with PDGFR: above (a and b) panels represents the 2D and 3D visualization of interactions of Avapritinib with PDGFR, where below (c and d) panels represents the 2D and 3D visualization of interactions of ZINC000002931631 with PDGFR.

In Fig. 9, it is evident that Avapritinib formed direct hydrogen bonds with LEU615; TYR676; SER917 and ASP973; while engaging in pi-stacking interactions with residues GLU675; ASP973; LEU615; LEU661; ARG585; TRP586, TYR676 and LYS623. Furthermore, van der Waals interactions involving important residues SER616 and ASP973 were observed. Whereas, ZINC000002931631 formed direct hydrogen bonds with ASP973; while engaging in pi-stacking interactions with residues GLY652; PRO653; SER972; LEU615; LEU660; LEU661; ARG585 and TRP586. Furthermore, van der Waals interactions involving important residues SER616; ARG585; THR649; MET648; ASN659; PHE969 and ASP973 were observed.

### Molecular dynamic simulations

To investigate the dynamic behavior of PDGFRA in its apo form, PDGFRA in complex with Avapritinib, and PDGFRA in complex with ZINC000002931631 we conducted three independent molecular dynamic simulations, each lasting 100 nanoseconds. Thus, a cumulative total of 300 nanoseconds of simulation data was generated for this study. In the first simulation, PDGFRA was simulated in its apo form. For the second simulation, we have taken the docked complex of PDGFRA in complex with Avapritinib. In the third simulation, we have taken the docked complex of PDGFRA in the complex with ZINC000002931631. These comprehensive molecular dynamic simulations aim to provide valuable information about the structural dynamics and interactions of PDGFRA in its apo state compared to in complex with the substrate ligands, shedding light on the impact of binding of Avapritinib compared to ZINC000002931631 as potential inhibitor of PDGFRA activity. The results of this study may have implications in understanding the molecular mechanisms underlying PDGFRA regulation and aid in the rational design of therapeutically relevant compounds targeting this enzyme.

### Analysis of PDGFRA dynamics during the simulated timescale

**Fig. 10:**
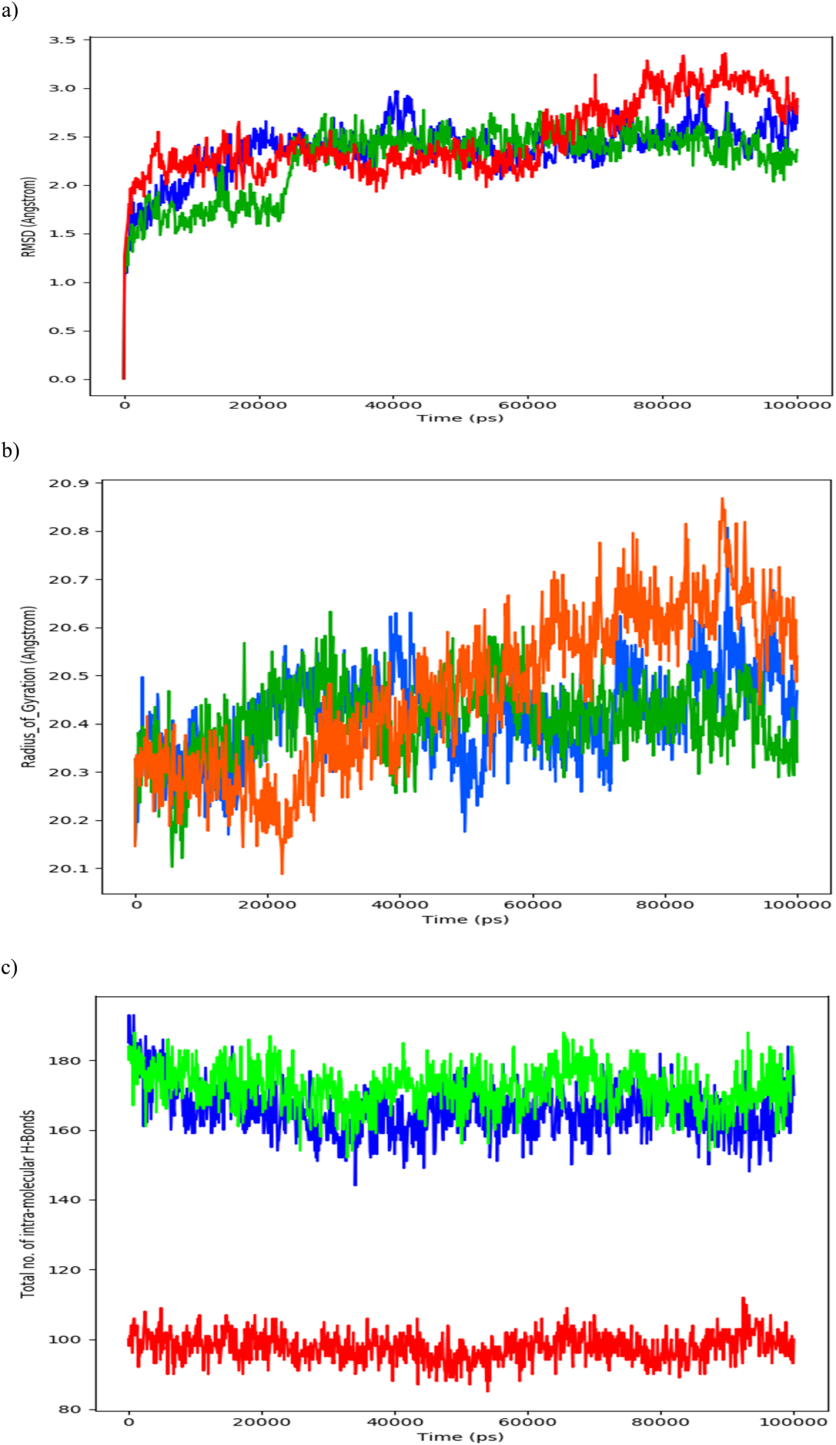
(a) Calculated PDGFRA protein's backbone RMSD in its apo state (RED), PDGFRA in complex with Avapritinib (BLUE), and PDGFRA in complex with ZINC000002931631 (GREEN). (b) Calculated PDGFRA protein's Radius of Gyration in its apo state (RED), PDGFRA in complex with Avapritinib (BLUE), and PDGFRA in complex with ZINC000002931631 (GREEN). (c) Calculated PDGFRA protein's total number of intramolecular hydrogen bonds in its apo state (RED), PDGFRA in complex with Avapritinib (BLUE), and PDGFRA in complex with ZINC000002931631 (GREEN).

**Fig. 11:**
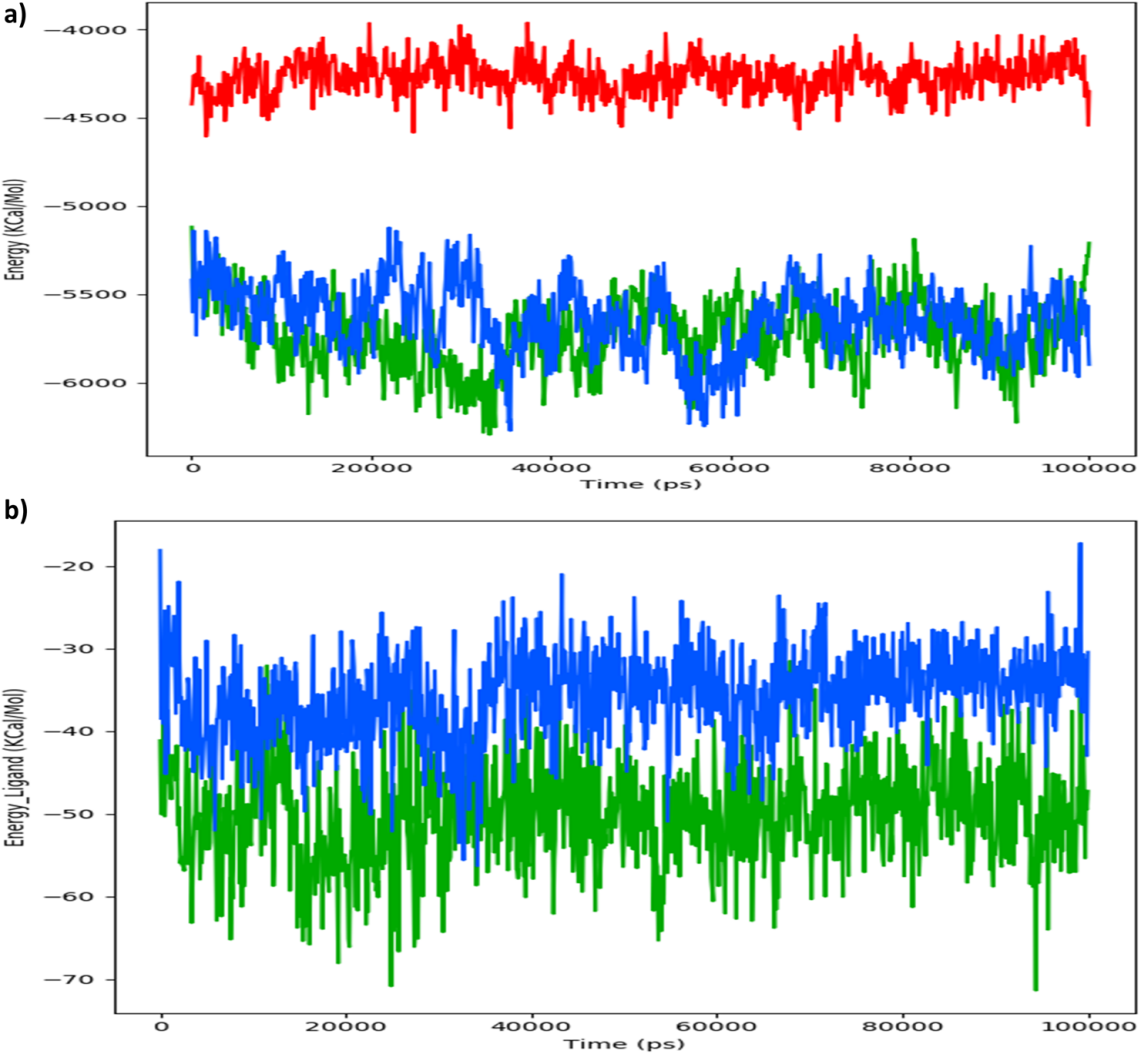
(a) Calculated PDGFRA protein's energy in its apo state (RED), PDGFRA in complex with Avapritinib (BLUE), and PDGFRA in complex with ZINC000002931631 (GREEN). (b) Calculated energy of ligand for PDGFRA in complex with Avapritinib (BLUE), and PDGFRA in complex with ZINC000002931631 (GREEN).

To comprehend the dynamics of PDGFRA, we conducted various analyses, including Root mean square deviation (RMSD) of the protein backbone (Fig. 10a), Radius of Gyration (ROG) of the protein (Fig. 10b), the total number of intramolecular hydrogen bonds within the protein (Fig. 10c), the energies (Fig. 11a and b) along with Secondary Structure Elements (SSE) impact with respect to the simulated timescale.

The protein's backbone RMSD fluctuated between 1.5 and 3.0 Å, with average values of 2.7, 2.5 and 2.2 Å for PDGFRA in its apo state, PDGFRA in complex with Avapritinib, and PDGFRA in complex with ZINC000002931631 at the binding site, respectively. Significant conformational changes in PDGFRA were observed during the initial and final 20 nanoseconds, particularly in the presence of the ZINC000002931631 compound complex at the binding site and in the apo state. These observations suggest that the ZINC000002931631 molecule induced conformational alterations in PDGFRA. Notably, at approximately 20 nanoseconds, a sudden increase in RMSD from 1.5 to 2.5 Å in the PDGFRA backbone was observed when complexed with the ZINC000002931631 molecule. Subsequently, the RMSD stabilized at around 2.5 Å with an average of 2.2 Å. It is interesting to note that the rise in PDGFRA RMSD, reaching up to 3.5 Å during the apo state simulation, was significantly attenuated when PDGFRA was complexed with Avapritinib and ZINC000002931631.

The protein's radius of gyration (ROG) exhibited fluctuations within the range of 20.1 to 20.9 Å. The average ROG values for PDGFRA in its apo state, PDGFRA in complex with Avapritinib, and PDGFRA in complex with ZINC000002931631 at the binding site were measured as 20.6 Å, 20.5 Å, and 20.4 Å, respectively. Notably, significant conformational changes in PDGFRA were observed during the initial and final 20 nanoseconds, particularly in the apo state. However, PDGFRA in combination with Avapritinib and ZINC000002931631 demonstrated relative stabilization, showing no significant fluctuations. These findings suggest that the flexibility of PDGFRA to undergo conformational changes is attenuated upon substrate molecule binding and stabilization within its binding pocket.

During the course of the simulation, notable variations in the total number of intramolecular hydrogen bonds were observed. These hydrogen bonds play a pivotal role in governing the rigidity of the protein, thereby influencing its ability to undergo conformational changes, which is crucial for the protein's activity. Specifically, a higher number of intramolecular hydrogen bonds generally leads to increased rigidity in the protein structure, limiting its flexibility to adopt different conformations. This flexibility is known to be of critical importance for the proper functioning of the protein in its specific biological context. In our simulations, PDGFRA in its apo state maintained an average of approximately 100 intramolecular hydrogen bonds throughout the simulated timescale. In contrast, when PDGFRA was complexed with Avapritinib and ZINC000002931631, the average number of intramolecular hydrogen bonds increased significantly, with Avapritinib maintaining an average of 170 hydrogen bonds and ZINC000002931631 maintaining an average of 180 hydrogen bonds. These findings suggest that the presence of Avapritinib and ZINC000002931631 in complex with PDGFRA induces an increase in intramolecular hydrogen bonding, potentially leading to a higher level of rigidity in the protein's structure, thus limiting its functionality. Such stabilization of the protein's conformation may have functional implications, as it could modulate the activity of PDGFRA in response to ligand binding.

In order to further validate the inhibitory potential of Avapritinib and ZINC000002931631 compounds targeting PDGFRA, we have investigated with particular focus on energy as a key parameter throughout the simulated timescale. Our analysis revealed that the average energy of the PDGFRA in its apo state during the simulation was approximately −4300 kcal mol^−1^. However, in the presence of Avapritinib, the PDGFRA exhibited a different energy profile, with the average energy maintaining approximately −5500 kcal mol^−1^. On the other hand, PDGFRA was found strongly inhibited by ZINC000002931631 as evident from its' much lowered energy with an approximate average of −5800 (Fig. 11a). Moreover, the binding energy profile of these compounds during the simulated timescale reveal that Avapritinib maintained an average of −35 Kcal mol^−1^ of binding energy, whereas ZINC000002931631 exhibited −55 Kcal mol^−1^ of binding energy (Fig. 11b).

These findings indicate that our proposed lead compound ZINC000002931631 has a notable strong inhibitory potential on PDGFRA and is very well comparable with FDA approved PDGFRA inhibitor Avapritinib in the least case scenario. The lower average energy observed in the presence of Avapritinib and ZINC000002931631 suggests that the binding of these compounds stabilize the PDGFRA conformation or affect its interactions with the surrounding environment. The altered energy landscape resulting from these compound bindings could be associated with potential functional implications, possibly influencing the functional activity or substrate binding capability of the PDGFRA. Further investigations are warranted to elucidate the precise mechanisms underlying the observed energy changes and their relevance to the enzymatic function of the PDGFRA. The analyses of RMSD, ROG, and intramolecular hydrogen bonds provided compelling evidence that specific conformational changes in the PDGFRA protein underlie the inhibitory potential of the Avapritinib and ZINC000002931631 compounds. However, the precise regions of the protein responsible for these inhibitory conformational changes remain uncertain. To address this question, we conducted a thorough investigation of the secondary structural elements (SSE) of PDGFRA throughout the simulated timescale.

**Fig. 12:**
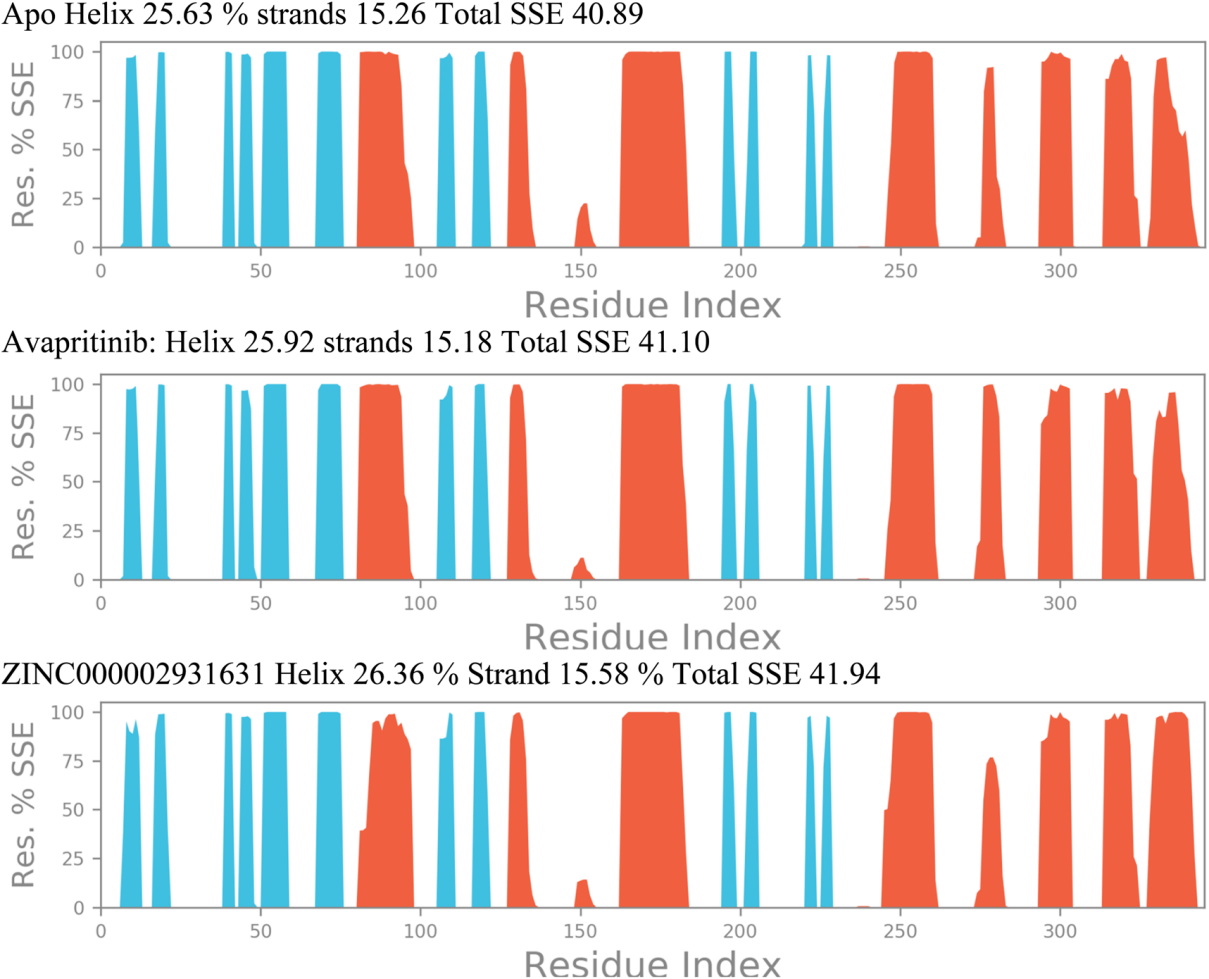
Calculated secondary structural elements (SSE) of PDGFRA protein's backbone in its apo state (top panel), PDGFRA in complex with Avapritinib (middle panel), and PDGFRA in complex with ZINC000002931631 (lower panel).

Fig. 12 illustrates the changes in a few alpha-helices and beta-sheets, particularly in regions adjacent to the initial 80–100, 150, and 270 residues. The significant changes observed in these SSEs suggest their involvement in the inhibitory action of the compounds. Notably, the overall SSE percentage increased from 40.89% in the apo state of the protein to 41.10% and 41.94% for PDGFRA in complex with Avapritinib and ZINC000002931631, respectively. These alterations in secondary structural elements are believed to be responsible for the observed fluctuations in RMSD, ROG, and intramolecular hydrogen bonds, particularly in the case of PDGFRA complexed with Avapritinib and ZINC000002931631 at the binding site. The conformational changes induced by these compounds seem to affect specific regions of the protein, leading to increased rigidity and inhibitory effects.

By shedding light on the dynamic changes in secondary structural elements of PDGFRA, our findings contribute to a deeper understanding of the molecular interactions and mechanisms underlying the inhibitory action of Avapritinib and ZINC000002931631 compounds on PDGFRA activity. These results may have implications for the design and development of novel targeted therapies against PDGFRA-related disorders. Further studies focusing on the specific interactions within the identified SSEs could provide valuable insights into the precise mode of inhibition and guide the rational design of more effective therapeutic agents.

### Molecular interactions between PDGFRA with Avapritinib and ZINC000002931631

**Fig. 13:**
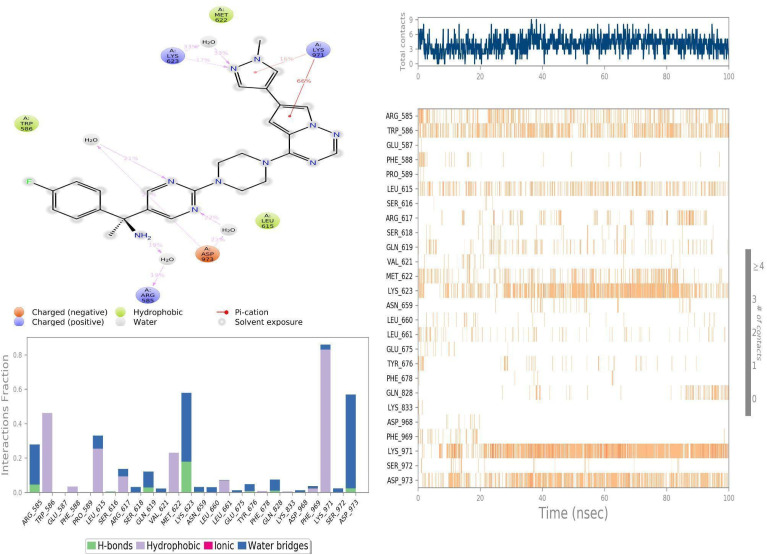
Molecular interactions observed between PDGFRA with Avapritinib during the simulated timescale.

To delve deeper, we subjected the docked PDGFRA-Avapritinib complex to simulation and examined the molecular level interactions responsible for the inhibitory effect. Throughout the simulation, Avapritinib formed upto 9 intermolecular contacts with several residues (Fig. 13). Notably, strong hydrophobic interactions with LYS971; TRP586; LEU615; MET622; ARG617; LEU661 and GLU587 was observed in order of their interaction strength. Hydrogen bonds with LYS623; GLN619; ARG585, TYR676 and ASP973 were noted. Among which, TYR676 and ASP973 residues were noted as critically important in stabilizing the PDGFRA-Avapritinib complex during the docking studies.

**Fig. 14:**
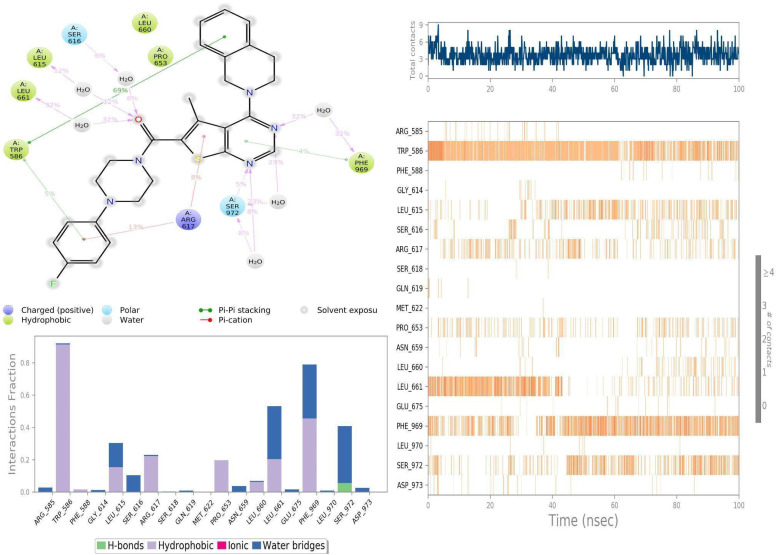
Molecular interactions observed between PDGFRA with ZINC000002931631 during the simulated timescale.

On the other hand, in the simulation where ZINC000002931631 was complexed with PDGFRA, the ZINC000002931631 compound was found to be tightly bound at the binding site throughout 100 ns of simulated timescale. Hydrophobic and water bridging interactions were observed as a crucial role player than direct hydrogen bonds (Fig. 14), which we believe to have led to conformational changes in the PDGFRA, especially near the binding site, possibly facilitating substrate binding. For example, TRP586 interacted with ZINC000002931631 for about 90% of the simulation time, and other residues, especially PHE969; LEU661; SER972, LEU615, ARG617 and PRO653, were found to interact with ZINC000002931631 for approximately 20% on an average of the simulation time. Among these, PHE969; SER972 and LEU661 residues formed water-mediated interactions with ZINC000002931631 for about 40% on average of the simulation time, respectively. Thus, in accordance with an earlier study that shows serine/threonine kinase has evolved to have large free-energy penalties (4–6 kcal mol^−1^) to adopt an inactive state relative to the active conformation when compared to tyrosine kinase (PDGFR and KIT). Suggesting challenges associated with designing type-I tyrosine kinase inhibitors in terms of selectivity. ^64^ Thus, such computational methods can help to design highly selective inhibitors for challenging drug targets.

## Conclusion

In this study, we employed a machine learning-assisted hybrid virtual screening strategy, which efficiently narrowed a library of 1.048 million compounds to 220 candidates with potential PDGFRA inhibitory activity—representing just 0.013% of the original library. To validate these candidates, we utilized the convolutional neural network-based *K*_DEEP_ algorithm for virtual screening, complemented by traditional genetic algorithm-based docking methods. Notably, ZINC000002931631 demonstrated comparable or even superior inhibitory potential against PDGFRA compared to Avapritinib, underscoring the robustness and utility of our machine learning approach. From docking and molecular dynamics simulation studies, we uncovered several key molecular–level interactions that are highly valuable for the design of target-specific inhibitors for the PDGFRA drug target. These findings highlight the potential of ZINC000002931631 as a promising candidate for further investigation. To translate our computational findings into practical therapeutic applications, we plan to undertake a series of experimental validations. Initially, *in vitro* assays will be conducted to evaluate Avapritinib's inhibitory activity on PDGFRA and KIT mutants, along with its effects on cellular proliferation and apoptosis in NSCLC cell lines. These studies will help establish its potential as a targeted therapy. Subsequently, *in vivo* assays using xenograft mouse models will be performed to assess its efficacy in reducing tumor growth and angiogenesis, as well as its ability to overcome resistance compared to existing tyrosine kinase inhibitors. This multi-stage experimental roadmap aims to rigorously validate the findings from this *in silico* study and advance them toward clinical applicability. As this work represents a preliminary computational study, we remain committed to thorough experimental follow-up to ensure the robustness and translational value of our approach.

## Data availability

All relevant data supporting the findings of this study are included within the manuscript. Any additional data or materials related to this work can be made available by the corresponding author upon reasonable request.

## Conflicts of interest

There are no conflicts to declare.
